# Cause-specific mortality in the general population with transient dipstick-proteinuria

**DOI:** 10.1371/journal.pone.0223005

**Published:** 2019-10-02

**Authors:** Kei Nagai, Kunihiro Yamagata, Kunitoshi Iseki, Toshiki Moriyama, Kazuhiko Tsuruya, Shouichi Fujimoto, Ichiei Narita, Tsuneo Konta, Masahide Kondo, Masato Kasahara, Yugo Shibagaki, Koichi Asahi, Tsuyoshi Watanabe

**Affiliations:** 1 University of Tsukuba, Tsukuba, Ibaraki, Japan; 2 The Steering Committee for “Design of the Comprehensive Health Care System for Chronic Kidney Disease (CKD) Based on the Individual Risk Assessment by Specific Health Checkups”, Tsukuba, Ibaraki, Japan; 3 Okinawa Heart and Renal Association, Okinawa, Japan; 4 Health Care Center, Osaka University, Suita, Japan; 5 Nara Medical University, Nara, Japan; 6 University of Miyazaki, Miyazaki, Japan; 7 Niigata University Graduate School of Medical and Dental Sciences, Niigata, Japan; 8 Yamagata University Graduate School of Medical Science, Yamagata, Japan; 9 Institute for Clinical and Translational Science, Nara Medical University Hospital, Nara, Japan; 10 St. Marianna University School of Medicine, Kawasaki, Kanagawa, Japan; 11 Iwate Medical University, Morioka, Japan; 12 Fukushima Rosai Hospital, Iwaki, Japan; Tokushima University Graduate school, JAPAN

## Abstract

Recently, changes in urinary albumin and in GFR have been recognized as risk factors for the development of end-stage kidney disease and mortality. Though most clinical epidemiology studies of chronic kidney disease (CKD) used renal function and proteinuria at baseline alone, definitive diagnosis of CKD with multiple measurements intensifies the differences in the risk for mortality between the CKD and non-CKD populations. We hypothesized that a transient diagnosis of proteinuria and reduced renal function each indicate a significantly higher mortality compared to definitive non-CKD as the negative control and lower mortality compared with definitive CKD as the positive control. The present longitudinal study evaluated a general-population cohort of 338,094 persons who received annual health checkups, with a median 4.3-year study period. There were 2,481 deaths, including 510 CVD deaths (20.6%) and 1,328 cancer deaths (53.5%), and mortality risk was evaluated for transient proteinuria and for transiently reduced renal function. The hazard ratios (HRs) for all-cause mortality and cancer mortality were not significant, but that for cardiovascular mortality was significantly higher for transient proteinuria (HR, 1.94 [95% confidence interval, 1.27–2.96] in men and 2.78 [1.50–5.16] in women). On the other hand, transiently reduced renal function was not significant for either cardiovascular mortality risk or cancer mortality risk. We surmise that this is the first study of the mortality risk of transient dipstick proteinuria in a large general-population cohort with cause-specific death registration. Transiently positive proteinuria appears to be a significant risk specifically for cardiovascular mortality compared with definitely negative for proteinuria.

## Introduction

Chronic kidney disease (CKD) is a risk factor for not only progression to end-stage kidney disease (ESKD), but also the development of cardiovascular disease (CVD) and all-cause mortality [1–7]. One recent cross-sectional study of a CKD population with CVD (34.7%) and malignant neoplasms (31.8%) as the leading causes of death demonstrated that a decline in the estimated glomerular filtration rate (eGFR) was associated with a higher risk of death due to CVD, but not malignancy [8]. Another study of a general population showed a slightly higher overall cancer mortality in CKD patients compared with non-CKD patients (adjusted hazard ratio [HR] 1.20 with a 95% confidence interval [CI] of 1.01–1.42) [9]. However, these investigations did not take variations in renal function and proteinuria into consideration.

Recently, the annual decline of the glomerular filtration rate (GFR) has been noted to be a risk factor for the development of ESKD and of CVD [10–14], and the change in eGFR is becoming a promising surrogate marker for clinical endpoints [10–17]. Moreover, the change in the albumin creatinine ratio (ACR) in urine is also considered a predictor for the incidence of CKD and of ESKD [18,19]. Compared to stable ACR, a 4-fold increase in ACR was associated with more than 1.5-times higher risk of all-cause mortality, CVD mortality and non-CVD mortality, respectively. It was also found that a 4-fold decrease in ACR seemed to be associated with higher CVD mortality (HR [95% CI], 1.35 [0.93–1.95]), while the HR for non-CVD mortality was not higher (0.89 [0.67–1.18]). These results were not significant for various possible reasons, but they implied that a change in the amount of urinary albumin or protein might be associated with cause-specific death during follow-up.

When calculating ΔeGFR, fluctuation of measured-creatinine values considerably affects transient decreases and increases in eGFR, and we should carefully categorize low-eGFR to avoid non-CKD by examining creatinine twice with an appropriate interval [20–23]. Moreover, persistent proteinuria by consecutive dipstick urinalysis should be used, because more than half of positive dipstick proteinuria turns negative in the general population [24]. Analyses with a definitive diagnosis of CKD intensified the differences in risk of advancing renal dysfunction and mortality between CKD and non-CKD populations [20–23]. However, the significance of a transient diagnosis (ie, false-positive) of CKD with consecutive assessments both of eGFR and of proteinuria was not fully demonstrated in a clinical epidemiology study with a general population-based cohort.

We hypothesized that a transient diagnosis of CKD indicates a significantly higher mortality compared to definitive non-CKD as the negative control and lower mortality compared with definitive CKD as the positive control. The present study evaluated a longitudinal, general population cohort of 338,094 persons who received annual health checkups, including more than two assessments of proteinuria and renal function, according to “The Specific Health Check and Guidance in Japan” program in 2008, and their course and cause of death, coded according to the International Classification of Diseases, 10th revision (ICD-10), were followed for a median of 4.3 years from the first measurement of eGFR. These analyses confirmed the significant risk of all-cause mortality and CVD mortality in definitive CKD with persistent proteinuria, with G3a in women or G3b in men, and further highlighted the CVD-specific mortality risk of transient proteinuria.

## Methods

### Study population

This longitudinal, cohort study was conducted according to the guidelines of the Declaration of Helsinki and was granted ethics approval by the relevant institutional review board (University of Tsukuba for ethical issues approved as No. 999, UMIN: 000019774). The study was performed as part of the prospective ongoing “Research on the Positioning of Chronic Kidney Disease in Specific Health Check and Guidance (so-called “Tokutei-Kenshin”) in Japan” project. Other details, such as the participants’ areas of residence, were reported previously [25]. The database was solely used and managed by the statistician, and the principal analyses to identify those of the screened subjects who died were completed by December 2018. Subsequent analyses were done using a standard analysis file (SAF) without any personal identifiers. The data included information about age, sex, body mass index (BMI), systolic blood pressure, diastolic blood pressure, smoking habit, and use of antihypertensive drugs, lipid-lowering drugs, and hypoglycemic drugs (obtained via self-reported questionnaire), the results of dipstick urinalysis for proteinuria and serological testing for serum creatinine concentration, and serum lipid status.

### Mortality surveillance

The underlying causes of death were coded according to ICD-10. Follow-up was conducted through December 2014. Cardiovascular mortality was defined as death due to circulatory diseases including myocardial infraction, heart failure, stroke, or sudden cardiac death reported as the 1st position cause of death (any ICD-10 code from the I chapter), as reported previously [19,25]. Cancer mortality was defined as death due to a neoplasm reported as C00-D48.

### Measurement of parameters

Urinalysis by the dipstick method was performed on a single spot-urine specimen. In Japan, the Japanese Committee for Clinical Laboratory Standards (http://jccls.org/) recommends that all urine dipstick results of 1+ correspond to a urinary protein level of 30 mg/dl; proteinuria was defined as 1+ or greater. In addition, blood samples were collected and assayed in an automatic clinical chemistry analyzer within 24 h of collection. Serum creatinine was measured using the enzymatic method. GFR was calculated using the formula of the Japanese Society of Nephrology [26]. CKD was defined as persistent positive proteinuria or reduced eGFR <60 ml/min/1.73 m^2^ both at the first measurement and the second measurement with an interval of more than one year. Hypertension was defined as systolic blood pressure ≥140 mm Hg and diastolic blood pressure ≥90 mmHg. Hypercholesterolemia was defined as low-density lipoprotein ≥140 mg/dl, high-density lipoprotein cholesterol ≤40 mg/dl, or triglycerides ≥200 mg/dl. These co-morbid conditions at the baseline year were used in the risk analysis.

### Statistical analysis

The primary outcomes for the analysis were all-cause, CVD, and cancer deaths during the follow-up period. Variables were age, sex, hypertension, proteinuria, low-density lipoprotein-cholesterol, high-density lipoprotein-cholesterol, triglycerides, cigarette smoking, use of antihypertensive medications, use of lipid-lowering drugs, and treatment for diabetes. The hypertension category was developed according to blood pressure levels (normal, <140/<90 mm Hg; hypertensive, ≥140/≥90 mm Hg) and use of antihypertensive medications. Mean- or median values and contingencies were first compared between definitive non-CKD (negative/negative for proteinuria and for reduced eGFR) and another category of CKD diagnosis, using Student’s *t*-test, Mann-Whitney’s test, the chi-squared test, or one-way ANOVA, as appropriate. Mean values and standard deviations are shown in each table. Hazard ratios (HRs) of the incidence of CVD by sex were estimated using the Cox regression model (SAS version 9.4, SAS Institute, Cary, NC, USA). A *P* value of <0.05 was considered significant.

## Results

**Fig 1:**
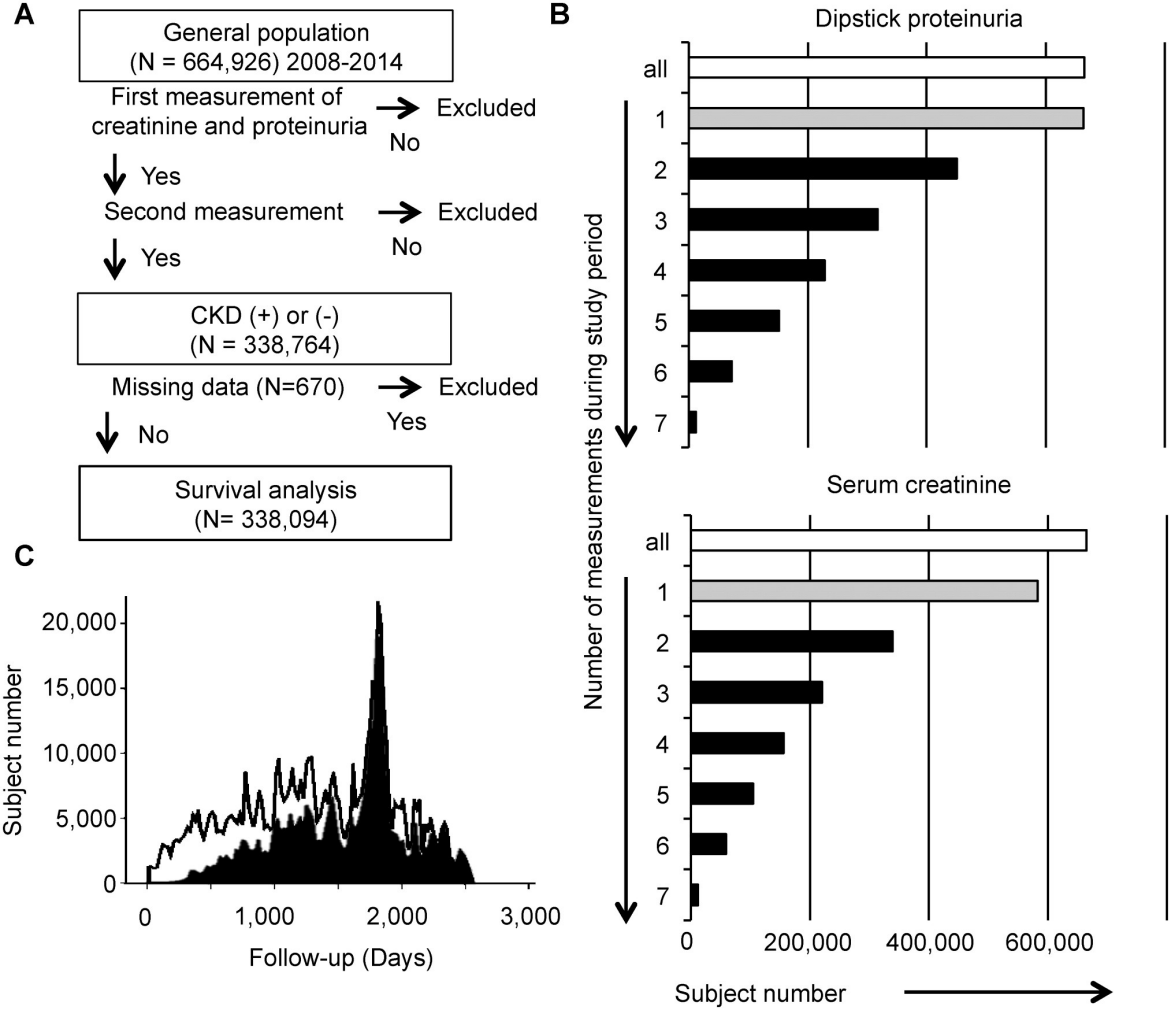
Study design. **A**. Strategy of recruitment of the study population, follow-up, and outcome analyses. Two annual measurements either of proteinuria or of serum creatinine enable definitive diagnosis of chronic kidney disease (CKD). **B**. Subject number having checked dipstick proteinuria and serum creatinine with various frequencies in this study population. The white bars mean all healthcheck participants, and the gray bars indicate their proportions with at least one measurement. Subjects with more than one check (ie, 2 to 7 times) represented by the black bars were recruited. **C**. The histograms present all healthcheck participants (black line) and the study population included in survival analyses (black filled). Abbreviations: CKD, chronic kidney disease.

A total of 664,926 subjects were screened, and the net subject population comprised 338,094 people (58.8% [n = 198,921] were women) who were aged from 40 to 74 years and for whom all of the data necessary for our research purposes were available (Fig 1A). Since the screened subjects had been examined with or without dipstick urinalysis and/or serum creatinine, 99.8% and 87.7% of screened subjects had at least one result for dipstick protein and eGFR, respectively. Similarly, 67.8% and 51.0% of the screened subjects had two or more results for them during the study period (Fig 1B). The study duration from the first measurement day of serum creatinine was 1 to 7 years (2008 through 2014, median 51 months, Fig 1C).

Table 1 shows the comparisons of baseline characteristics among the subpopulations having definitely negative proteinuria (-/- [first measurement/second measurement]), transient proteinuria (+/-), newly developed proteinuria (-/+), and definitive CKD with persistent proteinuria (+/+) in men and in women, respectively. The population having definitely negative proteinuria was the largest (88.6% in men and 94.0% in women), and their eGFR decrease was -0.4 ml/min/1.73 m^2^ between the first and second measurements. Subjects with transient proteinuria accounted for 3.8% of men and 2.3% of women, and they had older age, higher blood pressure, rate of using antihypertensive drugs and hypoglycemic drugs, worse dyslipidemia, rate of smoking, and BMI, and they had a lower eGFR. These characteristics were significant but milder than the subjects with persistent proteinuria.

**Table 1:** Demographics of the study population with and without definitive CKD determined by proteinuria.

		Men			
Proteinuria category	(first measurement) / (second measurement)	(-) / (-)	(+) / (-)	(-) / (+)	(+) / (+)
Study size	(persons)	123248	5347	5750	4828
Age	(years)	63 ± 8	63 ± 8**	64 ± 8***	65 ± 7***
Systolic blood pressure	(mmHg)	130 ± 17	135 ± 18***	134 ± 17***	139 ± 17***
Diastolic blood pressure	(mmHg)	78 ± 11	80 ± 11***	79 ± 11***	81 ± 11***
Use of antihypertensive drugs	(%)	30.9	44.3***	45.8***	60.5***
Use of hypoglycemic drugs	(%)	5.9	9.9***	11.5***	21.9***
Triglycerides	(mg/dl)	136 ± 99	149 ± 117***	150 ± 117***	165 ± 124***
Low-density lipoprotein	(mg/dl)	120 ± 30	120 ± 32	118 ± 31***	121 ± 32**
High-density lipoprotein	(mg/dl)	57.2 ± 15.1	56.2 ± 15.4***	55.6 ± 15.1***	54.2 ± 15.4***
Use of lipid-lowering drugs	(%)	10.4	13.2***	14.3***	20.6***
Smoking	(%)	24.4	27.7**	28.6***	29.1***
Body mass index	(kg/m^2^)	23.7 ± 3.0	24.4 ± 3.5***	24.3 ± 3.3***	25.2 ± 3.5***
Estimated GFR, first year	(ml/min/1.73 m^2^)	75.1 ± 15.3	73.0 ± 17.8***	73.8 ± 17.3***	65.9 ± 20.0***
Estimated GFR, second year	(ml/min/1.73 m^2^)	74.7 ± 15.3	73.3 ± 18.1***	71.7 ± 17.5***	63.7 ± 20.9***
		Women			
Proteinuria category	(first measurement) / (second measurement)	(-) / (-)	(+) / (-)	(-) / (+)	(+) / (+)
Study size	(persons)	186957	4521	4877	2566
Age	(years)	63 ± 8	64 ± 8***	64 ± 8***	65 ± 7***
Systolic blood pressure	(mmHg)	127 ± 17	132 ± 19***	132 ± 18***	137 ± 18***
Diastolic blood pressure	(mmHg)	75 ± 10	78 ± 16***	77 ± 11***	79 ± 11***
Use of antihypertensive drugs	(%)	26.0	40.5***	39.9***	56.3***
Use of hypoglycemic drugs	(%)	3.3	6.6***	7.4***	13.4***
Triglycerides	(mg/dl)	110 ± 64	120 ± 76***	120 ± 71***	143 ± 95***
Low-density lipoprotein	(mg/dl)	129 ± 30	130 ± 32*	129 ± 31	132 ± 32***
High-density lipoprotein	(mg/dl)	65.3 ± 15.8	63.2 ± 16.3***	63.4 ± 16.2***	60.8 ± 15.9***
Use of lipid-lowering drugs	(%)	18.6	23.0***	23.9***	30.2***
Smoking	(%)	5.0	6.1	6.8**	6.6
Body mass index	(kg/m^2^)	22.7 ± 3.3	23.9 ± 4.2***	23.7 ± 4.0***	25.0 ± 4.5***
Estimated GFR, first year	(ml/min/1.73 m^2^)	76.6 ± 15.9	74.2 ± 18.1***	75.4 ± 18.4***	67.5 ± 21.9***
Estimated GFR, second year	(ml/min/1.73 m^2^)	76.2 ± 15.9	74.6 ± 18.7***	73.5 ± 18.3***	65.7 ± 22.7***

Proteinuria was defined as 1+ or greater by dipstick tests.

*P<0.05,

**P<0.01, and

***P<0.001.

Abbreviations: CKD, chronic kidney disease; GFR, glomerular filtration rate.

Table 2 shows the comparisons of baseline characteristics among the subpopulations with definitely not reduced eGFR below 60 ml/min/1.73 m^2^ (-/-), transiently reduced eGFR (+/-), newly reduced eGFR (-/+), and definitive CKD with permanently reduced eGFR (+/+) in men and in women, respectively. The population with definitely not reduced eGFR was the largest (77.1% in men and 84.1% in women). The subjects with transiently reduced eGFR accounted for 4.7% of men and 4.0% of women, and they had older age, higher systolic blood pressure and rate of using antihypertensive drugs, worse dyslipidemia, higher BMI, and lower eGFR. These characteristics were significant but milder than the subjects with permanently reduced eGFR.

**Table 2:** Demographics of the study population with and without definitive CKD determined by eGFR.

		Men			
Reduced eGFR category	(first measurement) / (second measurement)	(-) / (-)	(+) / (-)	(-) / (+)	(+) / (+)
Study size	(persons)	107261	6596	7637	17617
Age	(years)	62 ± 9	66 ± 6***	65 ± 6***	67 ± 5***
Systolic blood pressure	(mmHg)	130 ± 17	131 ± 16*	133 ± 17***	133 ± 17***
Diastolic blood pressure	(mmHg)	78 ± 11	78 ± 10	79 ± 11***	79 ± 11***
Use of antihypertensive drugs	(%)	29.8	37.4***	40.3***	48.3***
Use of hypoglycemic drugs	(%)	6.4	6.6	7.9	9.2***
Triglycerides	(mg/dl)	137 ± 103	139 ± 97	145 ± 113***	141 ± 87***
Low-density lipoprotein	(mg/dl)	120 ± 30	122 ± 30***	120 ± 30	122 ± 30***
High-density lipoprotein	(mg/dl)	57.6 ± 15.3	56.1 ± 14.9***	55.3 ± 14.7***	54.2 ± 14.3***
Use of lipid-lowering drugs	(%)	9.7	13.1***	13.5***	17.4***
Smoking	(%)	26.9	17.3***	21.3***	16.0***
Body mass index	(kg/m^2^)	23.6 ± 3.1	24.1 ± 2.9***	24.1 ± 2.9***	24.3 ± 2.9***
Proteinuria, first year	(+ or more, %)	5.8	8.7***	9.4***	14.5***
Proteinuria, second year	(+ or more, %)	6.0	7.4	11.6***	14.8***
Estimated GFR, first year	(ml/min/1.73 m^2^)	79.9 ± 13.3	56.7 ± 4.3***	67.2 ± 5.8***	52.1 ± 7.2***
Estimated GFR, second year	(ml/min/1.73 m^2^)	79.5 ± 13.2	66.7 ± 5.6***	56.0 ± 4.4***	51.3 ± 7.8***
		Women			
Reduced eGFR category	(first measurement) / (second measurement)	(-) / (-)	(+) / (-)	(-) / (+)	(+) / (+)
Study size	(persons)	167328	7955	8796	14794
Age	(years)	63 ± 8	65 ± 6***	65 ± 6***	66 ± 6***
Systolic blood pressure	(mmHg)	127 ± 17	127 ± 17	130 ± 17***	130 ± 17***
Diastolic blood pressure	(mmHg)	75 ± 11	75 ± 10***	76 ± 10***	76 ± 10***
Use of antihypertensive drugs	(%)	25.5	30.6***	33.9***	38.8***
Use of hypoglycemic drugs	(%)	3.4	3.6	4.6	5.37***
Triglycerides	(mg/dl)	110 ± 64	115 ± 66***	119 ± 70***	121 ± 68***
Low-density lipoprotein	(mg/dl)	129 ± 30	133 ± 31***	130 ± 31**	131 ± 31***
High-density lipoprotein	(mg/dl)	65.5 ± 15.8	64.4 ± 16.0***	63.8 ± 15.8***	62.6 ± 15.4***
Use of lipid-lowering drugs	(%)	18.1	21.0**	22.2***	25.5***
Smoking	(%)	5.3	4.1	4.0**	3.7***
Body mass index	(kg/m^2^)	22.7 ± 3.4	23.0 ± 3.4***	23.3 ± 3.4***	23.4 ± 3.5***
Proteinuria, first year	(+ or more, %)	2.9	4.5***	4.5***	8.8***
Proteinuria, second year	(+ or more, %)	3.1	3.8	5.9***	8.9***
Estimated GFR, first year	(ml/min/1.73 m^2^)	80.1 ± 14.4	54.5 ± 4.6***	66.5 ± 6.5***	51.3 ± 6.6***
Estimated GFR, second year	(ml/min/1.73 m^2^)	79.7 ± 14.3	66.0 ± 6.5***	54.4 ± 4.1***	50.7 ± 7.1***

Outliers of eGFR with less than 5 ml/min/1.73 m^2^ (N = 110) were excluded. The criterion of reduced eGFR was set at less than 60 ml/min/1.73 m^2^.

*P<0.05,

**P<0.01 and

***P<0.001.

Abbreviations: CKD, chronic kidney disease; GFR, glomerular filtration rate.

There were 2,481 deaths, including 510 CVD deaths (20.6%) and 1,328 cancer deaths (53.5%) in the study period. When subdividing the subjects by proteinuria (Table 1) and by renal function (Table 2) with their variations between the first and second measurements, subjects with persistent proteinuria and with permanently reduced eGFR showed the highest or second highest mortality rate, as expected (Table 3). Particularly between transient proteinuria (+/-) and newly developed proteinuria (-/+), the predominance of CVD mortality in transient proteinuria and that of cancer mortality in newly developed proteinuria seemed to be counterbalanced.

**Table 3:** Crude all-cause and cause-specific mortalities of study subjects with and without definitive CKD.

Proteinuria category	(-) / (-)	(+) / (-)	(-) / (+)	(+) / (+)	
Men					
Number	123,248	5,347	5,750	4,828	
All-cause	1,368	70	88	118	
%	1.11	1.31	1.53	2.44	
Cardiovascular	255	24	18	35	
%	0.21	0.45	0.31	0.72	
Cancer	745	32	42	49	
%	0.60	0.60	0.73	1.01	
Women					
Number	186,957	4,521	4,877	2,566	
All-cause	755	26	37	19	
%	0.40	0.58	0.76	0.74	
Cardiovascular	147	11	9	11	
%	0.08	0.24	0.18	0.43	
Cancer	426	7	20	7	
%	0.23	0.15	0.41	0.27	
Reduced eGFR category	(-) / (-)	(+) / (-)	(-) / (+)	(+) / (+)	(Outliers)
Men					
Number	107,261	6,596	7,637	17,617	
All-cause	1,222	83	107	229	3
%	1.14	1.26	1.40	1.30	
Cardiovascular	233	22	16	61	0
%	0.22	0.33	0.21	0.35	
Cancer	645	42	62	119	0
%	0.60	0.64	0.81	0.68	
Women					
Number	167,328	7,955	8,796	14,794	
All-cause	667	36	36	98	0
%	0.40	0.45	0.41	0.66	
Cardiovascular	138	7	8	25	0
%	0.08	0.09	0.09	0.17	
Cancer	370	21	21	48	0
%	0.22	0.26	0.24	0.32	

Proteinuria was defined as 1+ or greater by dipstick tests. The criterion for reduced eGFR was set at less than 60 ml/min/1.73 m^2^. Excluding eGFR outliers (N = 110), three deaths could not be categorized. Abbreviations: eGFR, estimated glomerular filtration rate.

The HR for all-cause mortality was first examined in the total study population adjusted by age and sex (Table 4). Newly developed proteinuria had a higher risk for all-cause mortality following persistent proteinuria, and significance remained after adjustment with covariates possibly related to mortality. On the other hand, the mortality risk with transient proteinuria was weaker and disappeared after adjustment with covariates. Regarding CKD with permanently reduced eGFR, weaker risks for all-cause mortality were shown with and without multivariable-adjustment. Both in subjects with transiently reduced eGFR and those with newly reduced eGFR, there was no significant risk for all-cause mortality (Table 4).

**Table 4:** Adjusted hazard ratios for all-cause mortality in this study population with and without CKD.

Proteinuria	Age-, sex- adjusted model		Multivariable-adjusted	
Risk factor	HR	95% CI	HR	95% CI
Age, +1 year	1.07	1.07–1.08***	1.07	1.06–1.08***
Sex, women	0.37	0.34–0.40***	0.45	0.41–0.49***
Proteinuria, (+)/(-)	1.30	1.06–1.59*	1.22	0.99–1.50
Proteinuria, (-)/(+)	1.54	1.29–1.85***	1.42	1.19–1.70***
Proteinuria, (+)/(+)	2.23	1.87–2.65***	1.91	1.60–2.28***
Smoking, yes			1.74	1.58–1.92***
BMI, >25 kg/m^2^			1.03	0.94–1.13
Untreated HTN			1.15	1.03–1.29*
Treated HTN			1.15	1.03–1.28*
HTN with treatment			1.12	0.99–1.26
Hypertriglyceridemia			0.86	0.76–0.98*
High LDL			0.81	0.74–0.90***
Low HDL			1.46	1.28–1.68***
Use of lipid-lowering drugs			0.97	0.86–1.09
Use of hypoglycemic drugs			1.60	1.40–1.84***
Reduced eGFR	Age-, sex- adjusted model		Multivariable-adjusted	
Risk factor	HR	95% CI	HR	95% CI
Age, +1 year	1.07	1.07–1.08***	1.08	1.07–1.08***
Sex, women	0.36	0.33–0.39***	0.45	0.41–0.49***
Low eGFR, (+)/(-)	0.98	0.82–1.18	1.01	0.84–1.22
Low eGFR, (-)/(+)	1.12	0.94–1.33	1.11	0.93–1.31
Low eGFR, (+)/(+)	1.13	1.003–1.27*	1.13	1.004–1.28*
Smoking, yes			1.78	1.62–1.97***
BMI, >25 kg/m^2^			1.05	0.96–1.15
Untreated HTN			1.17	1.05–1.31**
Treated HTN			1.18	1.06–1.31**
HTN with treatment			1.17	1.04–1.32*
Hypertriglyceridemia			0.87	0.77–0.99*
High LDL			0.82	0.74–0.90***
Low HDL			1.46	1.27–1.68***
Use of lipid-lowering drugs			0.97	0.87–1.09
Use of hypoglycemic drugs			1.70	1.48–1.94***

Hazard ratios and 95% confidence intervals adjusted by age and sex, and by multiple variables, respectively. They are compared with definitely negative proteinuria (-)/(-) or with definitely not reduced eGFR (-)/(-) as the reference. The criterion of reduced eGFR was set at less than 60 ml/min/1.73 m^2^.

*P<0.05,

**P<0.01, and

***P<0.001.

Abbreviations: CKD, chronic kidney disease; HTN, hypertension; LDL, Low-density lipoprotein; HDL, High-density lipoprotein; eGFR, estimated glomerular filtration rate.

**Fig 2:**
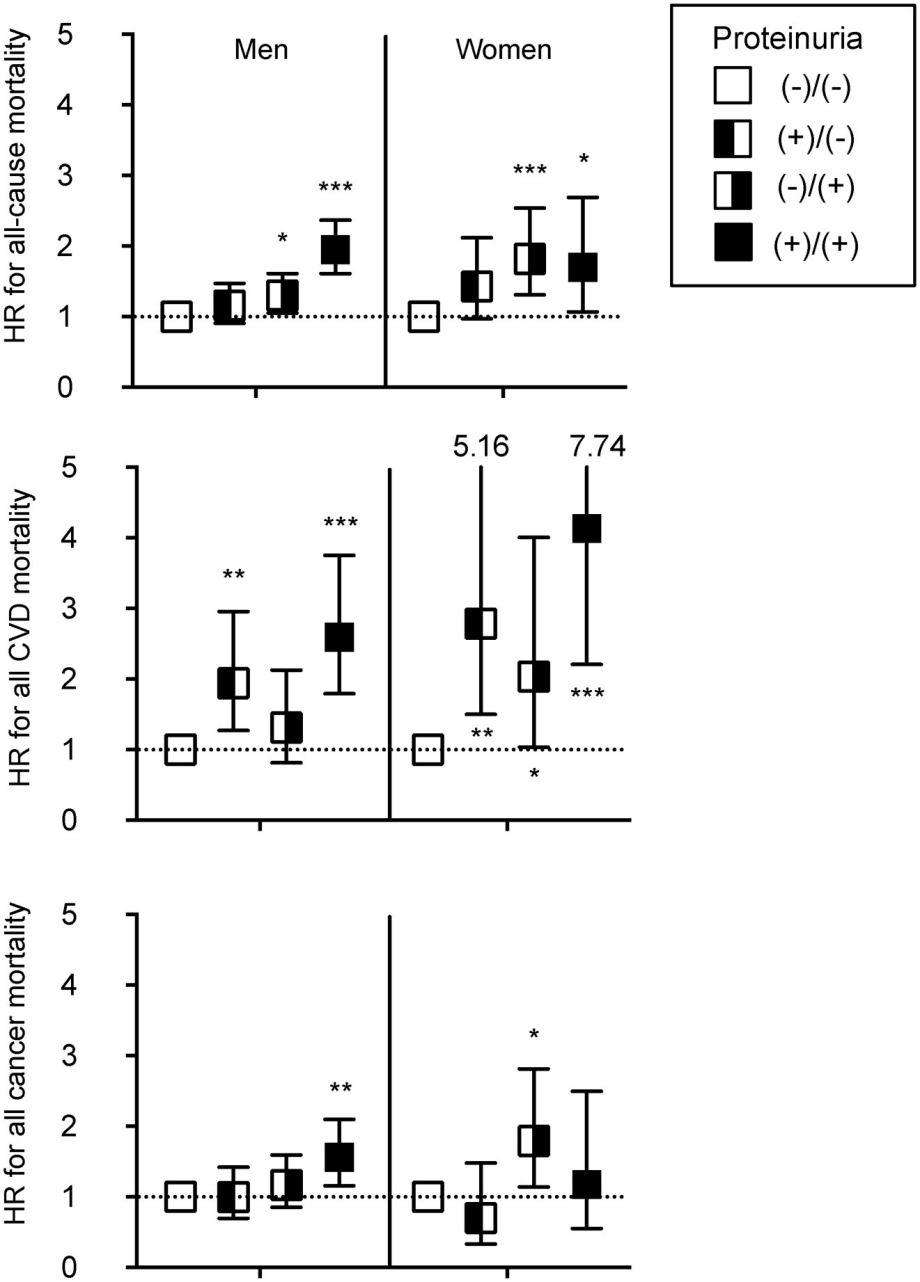
Multiply adjusted hazard ratios for cause-specific mortality with and without CKD determined by proteinuria. Symbol and error bars indicate multiply adjusted hazard ratios and 95% confidence intervals compared to definitely negative proteinuria (-)/(-) as the reference. Analyses are performed by sex; therefore, corresponding variables are similar to Table 4, except for sex. Abbreviations: CVD, cardiovascular diseases.

Fig 2 shows the HRs for all-cause mortality, CVD mortality, and cancer mortality, the leading causes of death in this study population. In men and in women, the HR for all-cause mortality was significant for newly developed proteinuria and for persistent proteinuria and not significant for transient proteinuria. The HR for CVD mortality was significantly higher for transient proteinuria (1.94 [95%CI 1.27–2.96] in men and 2.78 [1.50–5.16] in women) than for newly developed proteinuria (1.32 [0.81–2.13] in men and 2.04 [1.04–4.01] in women). On the other hand, the HR for cancer mortality for transient proteinuria was not significant and tended to be lower (0.99 [0.70–1.42] in men and 0.70 [0.33–1.48] in women) than that for newly developed proteinuria (1.17 [0.85–1.59] in men and 1.79 [1.14–2.82] in women).

**Fig 3:**
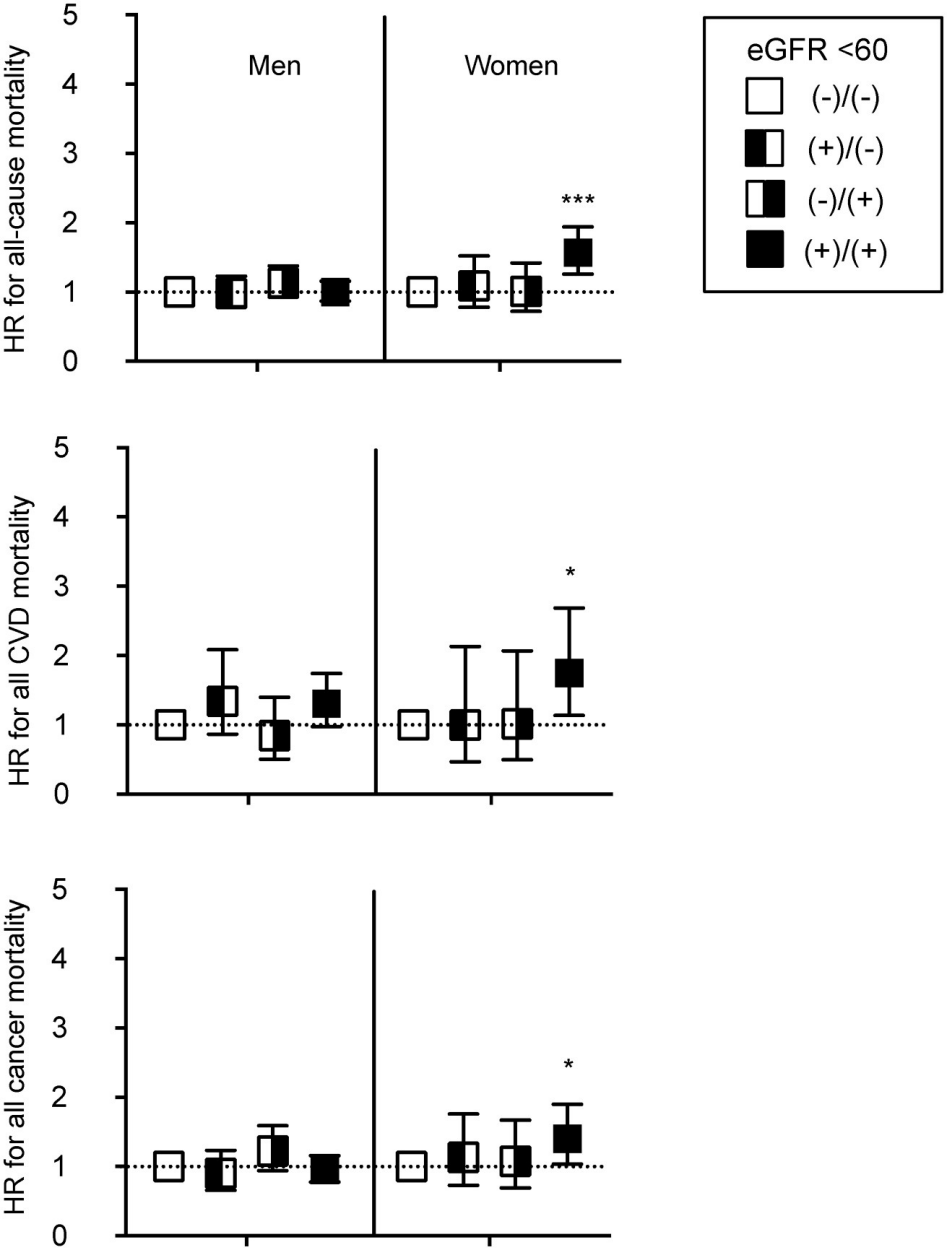
Multiply adjusted hazard ratios for cause-specific mortality with and without CKD determined by eGFR 60 ml/min/1.73 m^2^. Symbol and error bars indicate multiply adjusted hazard ratios and 95% confidence intervals compared to definitely without reduced eGFR (-)/(-) as the reference. Analyses are performed by sex, therefore, corresponding variables are similar to Table 4, except for sex. Abbreviations: CVD, cardiovascular diseases. eGFR, estimated glomerular filtration rate.

Regarding mortality risk with reduced eGFR, in women, the HR for all-cause mortality was significant with a permanently reduced eGFR less than 60 ml/min/1.73 m^2^, and it was not significant with a transiently reduced eGFR (Fig 3). In the same way, the HRs for CVD mortality and cancer mortality were significantly higher only with permanently reduced eGFR. Though there were no significant risks for all-cause mortality in men in Fig 3, a permanently reduced eGFR below 45 ml/min/1.73 m^2^ had a significant risk for all-cause mortality (S1 Fig). Moreover, a transiently reduced eGFR below 45 ml/min/1.73 m^2^ was not significantly associated with the risk for CVD mortality.

## Discussion

The present study demonstrated that a general population with transient dipstick proteinuria (1+ or more) had an increased risk for CVD mortality, while that with transiently reduced eGFR less than 60 ml/min/1.73 m^2^ did not have any increased risk for mortality in a longitudinal cohort of 338,094 persons from the general population with two or more assessments both of proteinuria and of renal function for dealing with metabolic syndrome as “Specific Health Check and Guidance”. As far as we know, there have been few attempts to assess cause-specific mortality related to changes in urinary albumin and protein levels in observational study settings. The Stockholm CREAtinine Measurements (SCREAM) project is a healthcare utilization study that contains 31,732 individuals with two or more ACR tests [19]. In this cohort, an ACR increase was associated with mortality, but the relationship was largely flat for an ACR decline. When focusing on cause-specific mortality, a 4-fold decrease in ACR tended to be associated with a higher risk for CVD mortality (HR [95%CI]; 1.35 [0.93–1.95]), while the HR for non-CVD mortality was not higher (0.89 [0.67–1.18]). This motivated us to examine the significance of transient proteinuria for CVD mortality in our large-sized study cohort.

Subjects with transient proteinuria or transiently reduced eGFR generally have worse co-morbid conditions (hypertension, use of hypoglycemic drugs, and lipid status) and worse physical status (higher BMI) compared to definitely negative proteinuria or definitely not reduced eGFR (Table 1), respectively. As a consequence, a higher crude-death rate was demonstrated (Table 3) comparing negative proteinuria or preserved eGFR, and adjusting for age and covariates was necessary to assess mortality risk (Table 4). Even if a prior study [19] and the present study were taken together, it is difficult to explain why decreases in urinary albumin and protein were involved in increased CVD mortality during the study follow-up period. One possibility as a cause for the higher CVD mortality risk with transient proteinuria compared to newly developed proteinuria may have been information that was not available in this study, such as prior development of proteinuria before 2008 at the starting year. However, this phenomenon does not conflict with the findings of prior randomized, controlled studies, because they compared placebo-adjusted treatment effects on the decline in albuminuria as clinical endpoints [27–29] that had comparable proteinuria levels at baseline, completely apart from the general population setting of the present study.

On the other hand, a decreased eGFR becomes a promising clinical endpoint because it can be a risk for the incidence of ESKD and the incidence and mortality of CVD [10–17]. Decline in eGFR in CKD and non-CKD patients should be assessed for the risk of incident and of all-cause mortality because the eGFR slope in CKD subjects with low-eGFR is much more significant and sensitive compared to that in subjects without low-eGFR [30]. Though the cut-off values of eGFR are flexible to assess the risk for mortality (<60 in Fig 3 and <45 ml/min/1.73 m^2^ in S1 Fig, were applied), as expected based on previous reports [10–14], newly reduced eGFR (-11.2 in men and -12.1 ml/min/1.73 m^2^ in women between two measurements) had a significant risk for all-cause mortality, as well as permanently decreased eGFR. Since some of these investigations also found a higher risk for mortality in a population with increased eGFR, it had been speculated that transiently reduced eGFR had a small risk for CVD mortality as well. However, at least there is no increased risk with transiently reduced eGFR compared to newly reduced eGFR, unlike in the context of proteinuria.

Transiently reduced eGFR might occur with some reasons, such as recovery from kidney injury, reduction of muscle mass, increased body fluid concentration due to participants’ fasting before blood test as well as transient proteinuria occurs. These mechanisms were supported by frequency of proteinuria decreased between first year and second year (8.7% to 7.4% in men and 4.5% to 3.8% in women, Table 2). However our current analyses could not conclude how come there is a different trend in risk for CVD mortality between in transient proteinuria and in transiently reduced eGFR. We further tried to elucidate risk in conjunction of transient proteinuria and transiently reduced eGFR (S2 Fig). Though number of CVD death was too small to make a conclusion, the result implied coincidence of transient proteinuria and reduced eGFR was also significant risk of CVD mortality but not non-CVD mortality (CVD; 3.12 [1.78–5.46] in men and 3.97 [1.46–10.76] in women).

As the scope of the present report was to elucidate the importance of transient proteinuria in cause-specific death, cancer mortality, as the leading cause of non-CVD death, was used just as the analytical control. Though the results shown in this article might partly contribute to promote the research field of cancer biology and epidemiology, more detailed analyses regarding the relationship between cancer and CKD have been in progress and will be reported by our group with a different methodology and a different study population from the screened subjects (*Tsuruya et al*. *in preparation*).

To the best of our knowledge, this is the first study to examine a large-sized (over 100,000 subjects) general population with both transient dipstick proteinuria and transiently reduced renal function and reliable death registration. Therefore, the strategy had the strength to perform sub-analyses of men or women enrolled and to detect cause-specific death. Moreover, dipstick proteinuria was routinely examined in the healthcheck, unlike ACR, which is not routinely tested with some healthcare policies. However, this study also has several limitations. First, the results of semi-quantitative dipstick proteinuria cannot be corrected by urinary creatinine and are affected by urinary concentration that might provide functional, pseudo-positive proteinuria. Second, the measurement of biochemical parameters including proteinuria and serum creatinine to determine subcategories was performed only once per year. Mortality rate in this cohort was not so high because of relatively shorter observational interval (4.2±1.3 years) and relatively younger population (63.2±7.8 years old at baseline). It was not possible to clarify what kind of specific intervention was performed in transient proteinuria from the first measurement, such as use of renin-angiotensin system inhibitors, which is necessary information to clarify the mechanisms of decreased proteinuria.

## Conclusion

From this study, transient proteinuria in the general population appears to be a significant risk factor specifically for CVD mortality, but not for non-CVD mortality. The association between transiently reduced eGFR and mortality is weaker, while permanently reduced eGFR clearly shows a higher HR for mortality, as expected. This appears to be the first study examining the mortality risk of transient dipstick proteinuria using a large-sized general population cohort with cause-specific death registration.

## Supporting information

S1 Fig Multiple-adjusted hazard ratios for all-cause mortality and cause-specific mortality with and without advanced CKD determined by eGFR 45 ml/min/1.73m^2^. Symbol and error bars indicate multiple variable-adjusted hazard ratios (HRs) and 95% confidence intervals (CIs) comparing to definitely without Low eGFR less than 45 ml/min/1.73m^2^ (-)/(-) as reference. Analyses were performed by sex, therefore, corresponding variables are similar to Table 4, except for sex. Abbreviations: CVD, cardiovascular diseases. eGFR, estimated glomerular filtration ratio.
(PDF)

S2 Fig Multiple variable-adjusted hazard ratios for all-cause mortality and cause-specific mortality with and without proteinuria and low eGFR. Symbol and error bars indicate multiple variable-adjusted hazard ratios (HRs) and 95% confidence intervals comparing to definitely without proteinuria or Low eGFR less than 60 ml/min/1.73 m2 (-)/(-) as reference. Analyses were performed by sex, therefore, corresponding variables are similar to Table 4, except for sex. Abbreviations: CVD, cardiovascular diseases. eGFR, estimated glomerular filtration ratio.
(PDF)
