# Supplementary material for: Cause-specific mortality in the general population with transient dipstick-proteinuria
Source: PLoS One. 2019 Oct 2;14(10):e0223005. doi: 10.1371/journal.pone.0223005 (PMC6774516; doi:10.1371/journal.pone.0223005)
Supplement: S1 Fig — Symbol and error bars indicate multiple variable-adjusted hazard ratios (HRs) and 95% confidence intervals (CIs) comparing to definitely without Low eGFR less than 45 ml/min/1.73m2 (-)/(-) as reference. Analyses were performed by sex, therefore, corresponding variables are similar to Table 4, except for sex. Abbreviations: CVD, cardiovascular diseases. eGFR, estimated glomerular filtration ratio. (PDF) [file pone.0223005.s001.pdf]

Supplemental Figure 1

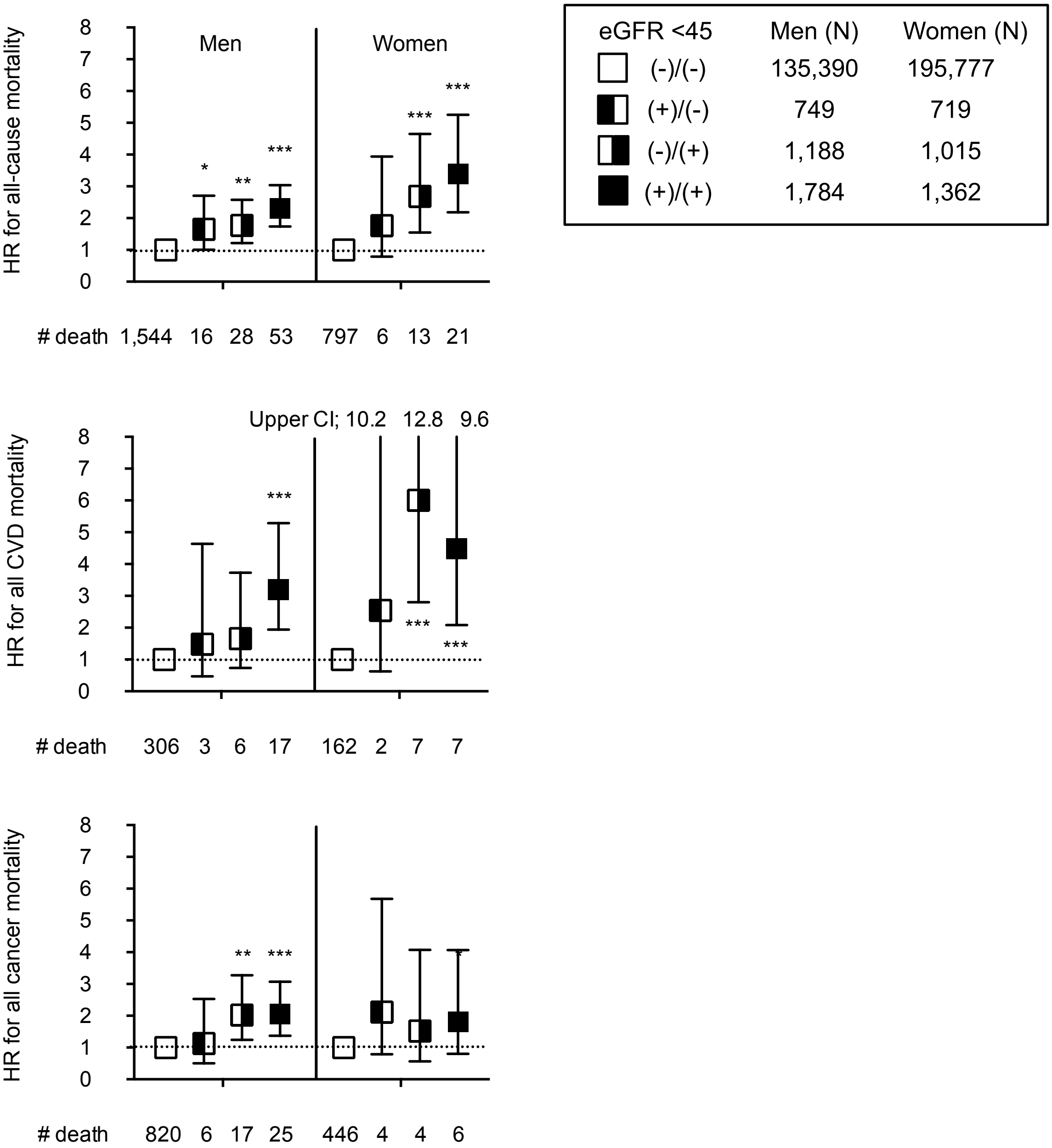

S1 Fig. Multiple variable-adjusted hazard ratios for all-cause mortality and cause-specific mortality with and without advanced CKD determined by eGFR 45 ml/min/1.73 m^2

Symbol and error bars indicate multiple variable-adjusted hazard ratios (HRs) and 95% confidence intervals (CIs) comparing to definitely without Low eGFR less than 45 ml/min/1.73 m^2 (-)/(-) as reference. Analyses were performed by sex, therefore, corresponding variables are similar to **Table 4**, except for sex.
Abbreviations: CVD, cardiovascular diseases. eGFR, estimated glomerular filtration ratio.
